# Multi‐cohort evaluation of plasma *p*‐tau217+ classification accuracies and thresholds for early detection of amyloid‐beta pathology

**DOI:** 10.1002/alz70856_104953

**Published:** 2026-01-07

**Authors:** Wasiu Gbolahan Balogun, Gallen Triana‐Baltzer, Anum Saeed, Xuemei Zeng, Alexandra Gogola, Brian J Lopresti, Victor L. Villemagne, Mary Ganguli, Hartmuth C. Kolb, Beth E. Snitz, Oscar L Lopez, Ann D Cohen, Steven E. Reis, Thomas K Karikari

**Affiliations:** ^1^ University of Pittsburgh, Pittsburgh, PA, USA; ^2^ Neuroscience Biomarkers, Janssen Research & Development, LLC, San Diego, CA, USA; ^3^ University of Pittsburgh School of Medicine, Pittsburgh, PA, USA; ^4^ University of Pittsburgh, School of Medicine, Pittsburgh, PA, USA; ^5^ University of Pittsburgh School of Public Health, Pittsburgh, PA, USA; ^6^ Division of Geriatrics & Neuropsychiatry, Department of Psychiatry, University of Pittsburgh, Pittsburgh, PA, USA

## Abstract

**Background:**

Blood biomarkers represent the next generation of Alzheimer's disease (AD) diagnostics, enabling noninvasive, inexpensive, and scalable monitoring of amyloid‐beta (Aβ) plaque (A) and tau neurofibrillary tangles (T) pathologies and neurodegeneration (N). Plasma *p*‐tau217 has emerged as perhaps the most promising AD blood biomarker, prompting the development of several technologies to evaluate its prognostic and diagnostic utility. However, cross‐cohort validation studies in community‐based cohorts are lacking.

**Methods:**

Here, we assessed the Janssen plasma *p*‐tau217+ assay in three community‐based cohorts: the Monongahela Youghiogheny Healthy Aging Team‐Neuroimaging (MYHAT‐NI) with 113 participants (Aβ‐PET positivity=24.8%), the Human Connectome Project (HCP) comprising 234 participants (Aβ‐PET positivity= 15.0%) and the Heart Strategies Concentrating on Risk Evaluation study (Heart SCORE) made up of 154 participants (Aβ‐PET positivity=18.2%), all recruited from southwestern Pennsylvania, USA. We employed [^11^C] Pittsburgh Compound B (PiB) positron emission tomography (PET) imaging for brain Aβ load. We utilized receiver operating characteristic (ROC) curves to evaluate *p*‐tau217+ accuracies in detecting Aβ pathology, adjusting for age, sex, and APOE4 carrier status.

**Results:**

The Janssen *p*‐tau217+ assay exhibited high performance in identifying Aβ PET positivity in all three cohorts, with AUCs of 91% for MYHAT‐NI, 93% for HCP, and 82% for Heart SCORE. Plasma *p*‐tau217+ showed high specificity: MYHAT‐NI (57%), HCP (81%), Heart SCORE (87%) but poor sensitivity to Aβ PET: MYHAT‐NI (75%), HCP (70%), Heart SCORE (59%). *p*‐tau217+ was strongly correlated with Aβ‐PET SUVR and was stronger in the Aβ‐PET‐positive sub‐groups MYHAT‐NI (*r* = ‐0.1292; *p* = 0.2443), HCP (*r* = ‐0.1617; *p* = 0.0347), Heart SCORE (*r* = 0.1586; *p* = 0.0896). Importantly, correlation with Aβ‐PET was strongest in MYHAT‐NI which had the highest proportion of Aβ‐PET‐positive participants.

**Conclusions:**

The Janssen *p*‐tau217+ assay identifies Aβ pathology in cognitively normal older adults in the community, underscoring its potential utility as a diagnostic tool for investigating AD at the population‐level.

